# Larotrectinib efficacy for liver metastases in papillary thyroid carcinoma patient harboring *SQSTM1–NTRK1* fusion

**DOI:** 10.1186/s40792-024-01971-1

**Published:** 2024-07-17

**Authors:** Haruhiko Yamazaki, Makoto Sugimori, Aya Saito

**Affiliations:** 1 Department of Breast and Thyroid Surgery, Yokohama City University Medical Center, 4-57 Urafunecho, Minami-Ku, Yokohama, Kanagawa 232-0024 Japan; 2 Division of Cancer Genome Medicine, Genomics Laboratory, and Gastroenterology, Yokohama City University Medical Center, 4-57 Urafunecho, Minami-Ku, Yokohama, Kanagawa 232-0024 Japan; 3 Department of Surgery, Yokohama City University School of Medicine, 3-9 Fukuura, Kanazawa-Ku, Yokohama, Kanagawa 236-0004 Japan

**Keywords:** Larotrectinib, *NTRK* fusion, Papillary thyroid carcinoma

## Abstract

**Background:**

Pooled data analysis from three phase I/II larotrectinib clinical trials revealed that larotrectinib demonstrated rapid and durable disease control and a favorable safety profile for patients with neurotrophic-tropomyosin receptor kinase (*NTRK*) fusion positive thyroid carcinoma. Herein, we report the case of a patient with papillary thyroid carcinoma (PTC) and liver metastases who demonstrated a durable response to treatment with larotrectinib.

**Case presentation:**

A 50-year-old female with PTC was referred to our hospital for postoperative observation. Computed tomography (CT) scan was performed to screen for distant metastasis, since thyroglobulin concentration increased gradually, and revealed multiple distant metastases, including multiple liver metastases. Radioactive iodine was administered at a dose of 100 mCi. However, uptake was observed only in the thyroid bed, and distant metastases had no avidity. As liver metastases progressed, lenvatinib (24 mg/day) was initiated after confirmation of liver metastases by liver biopsy 9 years and 1 month after the initial referral to our hospital. Since the multiple metastases became refractory for lenvatinib, the OncoGuide™ NCC Oncopanel System was performed, and the *SQSTM1–NTRK1* gene fusion was confirmed. Larotrectinib was subsequently administered at a dose of 200 mg/day. The CT before the initiation of larotrectinib showed multiple liver metastases with a maximum diameter of 48 mm. The first CT evaluation at 1 month after the initiation of larotrectinib treatment showed that the tumor volume was reduced by 28% in the RECIST 1.1 criteria. After 3 months of larotrectinib treatment, a 38% reduction in the tumor volume was achieved as the best clinical response. The only side effect was grade 1 myalgia. At 12 months after the initiation of larotrectinib treatment, none of the lesions had progressed.

**Conclusions:**

In conclusion, larotrectinib demonstrated effective antitumor activity against liver metastases of PTC, a relatively rare site of distant metastasis. Furthermore, the efficacy of larotrectinib was maintained, even though the patient had a history of multi-tyrosine kinase inhibitor treatment and a relatively infrequent fusion gene, *SQSTM1–NTRK1*.

## Background

Papillary thyroid carcinoma (PTC) is the most common histological type of thyroid carcinoma [1]. In general, almost all PTCs have relatively good prognoses. However, some patients with PTC develop distant metastases [2]. Subsequently, multi-tyrosine kinase inhibitors (mTKIs), such as sorafenib and lenvatinib, have been used to treat progressive radioiodine-refractory PTCs [3, 4]. The most frequent target lesions in TKI treatment are the lungs, lymph nodes, and bone [5]. In contrast, liver metastasis from PTC is rare [6].

The identification of pathways involved in the pathophysiology of carcinogenesis, metastasis, and drug resistance, as well as the emergence of technologies enabling the molecular analysis of tumors and the discovery of targeted therapies, has stimulated research focusing on the optimal use of targeted agents [7]. Cancer genomic medicine (CGM), which optimizes therapeutic interventions based on tumor genome profiling, has demonstrated clinical benefits in a wide range of cancer subtypes [8]. Japan started CGM in 2019 with two approved comprehensive genomic profiling (CGP) tests under the national health insurance umbrella [8]. CGP is appropriate for patients who have completed or are nearing the completion of standard treatment for thyroid cancers with locally advanced or metastatic disease. Larotrectinib was approved in Japan in 2021 for advanced or recurrent solid tumors harboring neurotrophic-tropomyosin receptor kinase (*NTRK*) fusions. Larotrectinib is an orally administered ATP-competitive inhibitor of TRKA, TRKB, and TRKC [9]. Pooled data analysis from three phase I/II larotrectinib clinical trials (NCT02576431, NCT02122913, and NCT02637687) included 29 patients with TRK-fusion-positive thyroid cancer. The analysis revealed that larotrectinib demonstrated rapid and durable disease control and a favorable safety profile [10].

Herein, we report the case of a patient with PTC and liver metastases who demonstrated a durable response to treatment with larotrectinib, along with a review of the relevant literature.

## Case presentation

A 50-year-old female with PTC was referred to our hospital for postoperative observation. She had previously undergone a subtotal thyroidectomy and right-sided modified neck dissection for PTC. She developed local recurrence in the remnant right lobe of the thyroid gland, and completion thyroidectomy was performed 4 years and 6 months after her initial referral to our hospital. Three years after surgery for local recurrence, a computed tomography (CT) scan was performed to screen for distant metastasis, since the thyroglobulin (Tg) concentration increased gradually. Computed tomography (CT) revealed mediastinal lymph node, lung, left adrenal gland, splenic, and multiple liver metastases. Radioactive iodine (RAI) was administered at a dose of 100 mCi. However, uptake was observed only in the thyroid bed, and distant metastases had no avidity. Six months after the first RAI treatment, the patient underwent a whole-body scan, which showed uptake only in the thyroid bed and not at the sites of distant metastasis. Therefore, we considered the sites of distant metastases to be refractory to RAI. As liver metastases progressed, lenvatinib (24 mg/day) was initiated after confirmation of liver metastases by liver biopsy 9 years and 1 month after the initial referral to our hospital. The patient’s blood test results before the initiation of lenvatinib were as follows: aspartate aminotransferase (AST), 16 U/L; alanine aminotransferase (ALT), 14 U/L; creatinine (Cre), 0.64 mg/dL; blood urea nitrogen (BUN), 10.0 mg/dL; thyroid-stimulating hormone (TSH), 0.269 μIU/mL; free triiodothyronine (F-T3), 2.54 pg/mL; free thyroxine (F-T4), 1.39 ng/dL; Tg, 635 ng/mL; and Tg antibody (TgAb), 13 IU/mL. The CT before the initiation of lenvatininb showed multiple liver metastases with a maximum diameter of 30 mm. Although the liver metastases shrank due to lenvatinib treatment and the Tg concentration reached a nadir of 26.0 ng/mL, the medication was changed to sorafenib (400 mg/day) due to grade 3 proteinuria. After that, lenvatinib (10 mg/day) was restarted due to progressive disease under sorafenib treatment. However, the multiple metastases became refractory for lenvatinib.

Since we considered that the previous tissue specimens from the liver biopsy may not be suitable for genomic testing due to deterioration, we performed another liver biopsy. First, the Oncomine Dx Target Test (Thermo Fisher, USA) was performed to screen for *RET* mutations. The sampled liver tissue was negative for *RET* mutations. In addition, *BRAF* mutations were not detected. However, a *SQSTM1–NTRK1* fusion was identified. Then, the OncoGuide™ NCC Oncopanel System (Sysmex Co., Ltd., Kobe, Japan) was performed to confirm the *SQSTM1–NTRK1* gene fusion (*SQSTM1* [chr5:179,256,832;hg19/GRCh37] joining with *NTRK1* [chr1:156,843,753;hg19/GRCh37]). No other pathogenic mutations were identified.

**Fig. 1:**
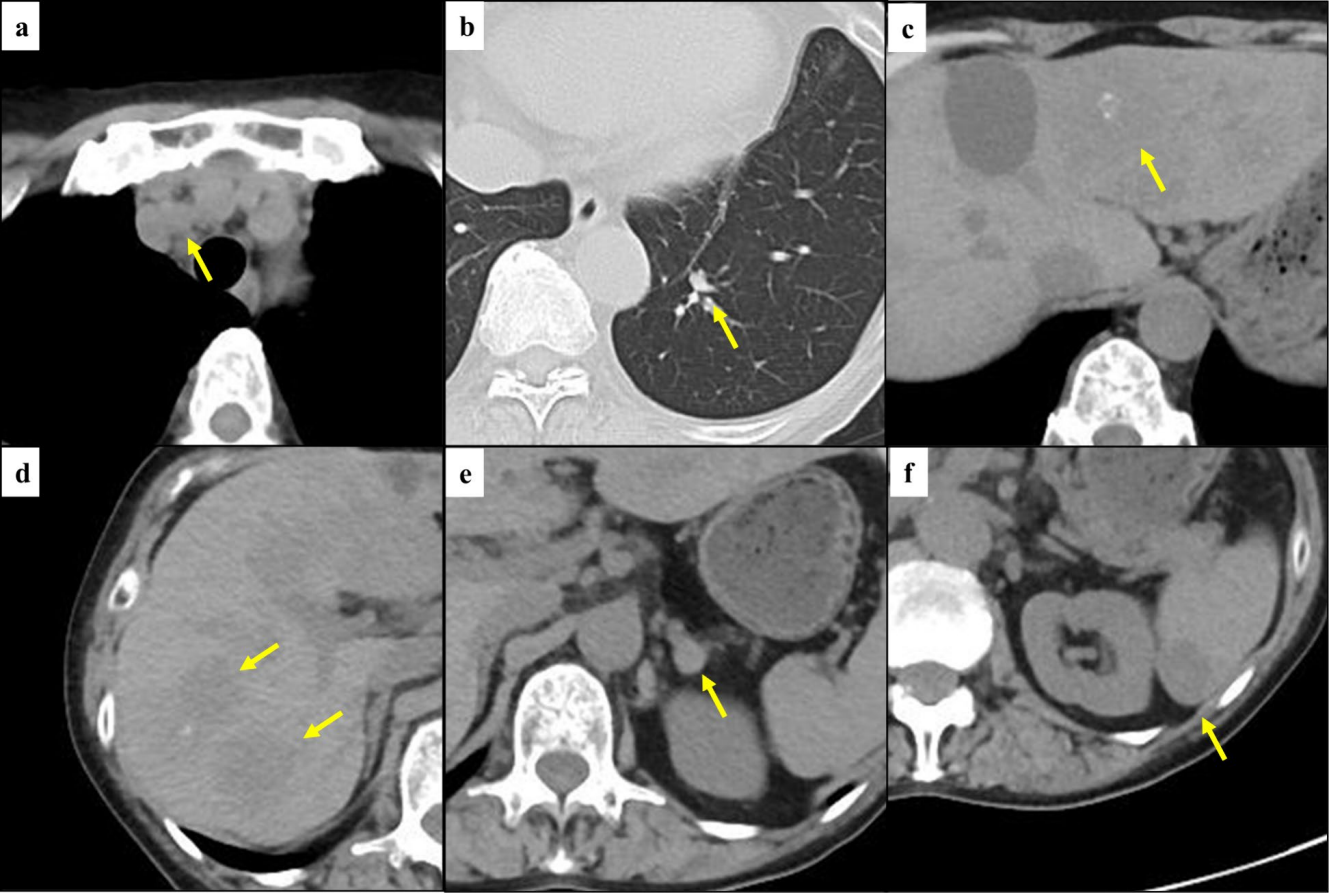
Computed tomography before larotrectinib initiation. The computed tomography before larotrectinib initiation showed mediastinal lymph node (**a**), lung (**b**), liver (**c**, **d**), left adrenal gland (**e**), and splenic (**f**) metastases

**Fig. 2:**
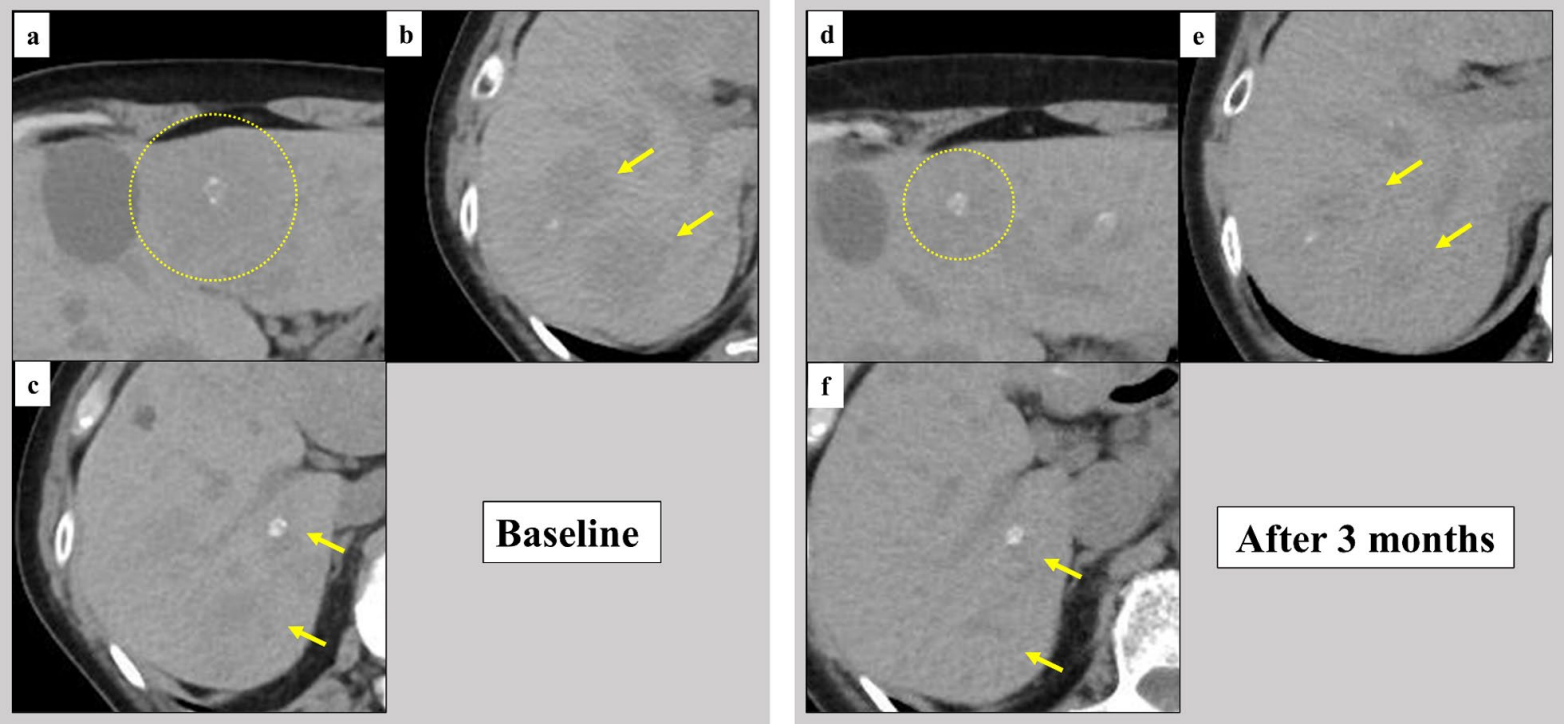
Baseline and 3 months after larotrectinib computed tomography images. The computed tomography before the initiation of larotrectinib showed multiple liver metastases with a maximum diameter of 48 mm (**a**–**c**). After 3 months of larotrectinib treatment, a 38% reduction in the tumor volume was achieved as the best clinical response (**d**–**f**)

After receiving approval from the expert panel, the decision was made to initiate larotrectinib. Larotrectinib was subsequently administered at a dose of 200 mg/day. The patient’s blood test results before the initiation of larotrectinib were as follows: AST, 12 U/L; ALT, 14 U/L; Cre, 2.08 mg/dL; BUN, 45.0 mg/dL; TSH, < 0.005 μIU/mL; F-T3, 3.13 pg/mL; F-T4, 1.80 ng/dL; Tg, 9960 ng/mL; and TgAb, < 10 IU/mL. Since patient had renal dysfunction due to proteinuria by adverse effect of lenvatinib, an enhanced CT could not be performed. The CT before the initiation of larotrectinib showed multiple liver metastases with a maximum diameter of 48 mm, and lung, mediastinal lymph node, spleen, and left adrenal gland metastases were suspected (Figs. 1 and 2a–c).

**Fig. 3:**
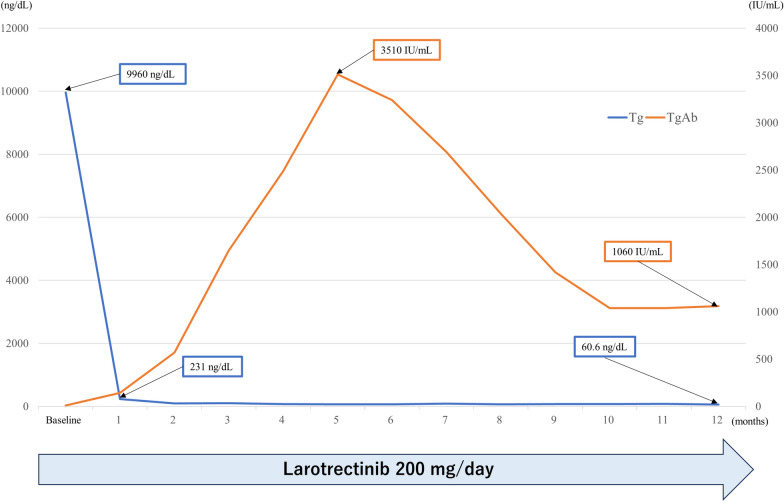
Clinical course after larotrectinib. The thyroglobulin (Tg) and Tg antibody (TgAb) concentrations were 635 ng/mL and 13 IU/mL, respectively. The nadir of the Tg concentration was 62.9 ng/mL during larotrectinib treatment, but the TgAb level gradually increased to 3510 IU/mL. Subsequently, the TgAb level gradually decreased. During larotrectinib treatment, thyroid-stimulating hormone level had been suppressed to < 0.005 μIU/mL

The first CT evaluation at 1 month after the initiation of larotrectinib treatment showed that the tumor volume was reduced by 28% in the RECIST 1.1 criteria [11]. After 3 months of larotrectinib treatment, a 38% reduction in the tumor volume was achieved as the best clinical response (Fig. 2d–f). The nadir of the Tg concentration was 62.9 ng/mL during larotrectinib treatment, but the TgAb level gradually increased to 3510 IU/mL. Subsequently, the TgAb level gradually decreased (Fig. 3). During larotrectinib treatment, TSH level had been suppressed to < 0.005 μIU/mL. The only side effect was grade 1 myalgia. At 12 months after the initiation of larotrectinib treatment, none of the lesions had progressed.

## Discussion

A rearrangement involving one of the NTRK genes, which belongs to the NTRK family, represents a significant oncogenic event in thyroid cancer [12]. The NTRK family includes three genes, *NTRK1*, *NTRK2*, and *NTRK3*, which encode TRKA, B, and C, respectively. Fusions involving the kinase domain of the *NTRK* gene lead to uncontrolled activation of the tyrosine receptor kinase and, subsequently, the MAPK, PI3K/AKT, and PLC pathways [13]. TRK fusion-positive thyroid carcinoma is more commonly associated with a younger age at diagnosis, and approximately 5–25% of pediatric PTC cases are reported to harbor *NTRK* gene fusions [10]. In contrast, the reported presence of *NTRK* fusions in predominantly adult thyroid cancer cohorts is low, ranging from 2.3 to 3.4% [14]. To date, no large-scale study has investigated the incidence of *NTRK* fusions in Japanese patients with thyroid cancer. As genomic testing has become increasingly common in Japanese clinical practice, the incidence of *NTRK* fusion in Japanese patients with thyroid cancer is likely to become clear in the near future.

Several *NTRK* rearrangements have been observed in TCs. Chu et al. investigated 351 primary thyroid cancers, including 186 PTCs, 54 medullary thyroid carcinomas (MTCs), 38 anaplastic thyroid carcinomas (ATCs), 24 Hurthle cell carcinomas (HCCs), 23 follicular thyroid carcinomas (FTCs), 21 poorly differentiated thyroid carcinomas (PDTCs), and five unclassifiable carcinomas [15]. The study reported that *NTRK* rearrangements were detected in 11 (3.1%) cases, all of which were originally diagnosed with PTC [15]. The observed *NTRK* rearrangements included *ETV6–NTRK3* (n = 4), *TPR–NTRK1* (n = 2), *RBPMS–NTRK3* (n = 2), *SQSTM1–NTRK1* (n = 1), *SQSTM1–NTRK3* (n = 1), and *EML4–NTRK3* (n = 1) [15]. In another study by Pekova et al., a total of 989 thyroid cancer tissues, including 851 PTCs, 86 MTCs, 19 FTCs, 13 ATCs, 10 PDTCs, 10 HCCs, and 30 borderline thyroid tumor tissues (17 follicular tumors of uncertain malignant potential, 13 non-invasive follicular tumors with papillary-like nuclear features, and 194 benign thyroid tissues) were analyzed [12]. The analyses revealed the *NTRK* fusion gene in 59 patients with thyroid cancer, 57 of 846 (6.7%) patients with PTC, and 2 of 10 (20.0%) patients with PDTC [12]. In addition, eight types of *NTRK* fusions were identified: E*TV6–NTRK3* (n = 38), *TPM3–NTRK1* (n = 5), *SQSTM1–NTRK3* (n = 4), *EML4–NTRK3* (n = 4), *RBPMS–NTRK3* (n = 3), *IRF2BP2–NTRK1* (n = 3), *SQSTM1–NTRK1* (n = 1), and *TPR–NTRK1* (n = 1) [12]. *SQSTM1–NTRK1* was also detected in this case. Considering previous reports, the *SQSTM1–NTRK1* fusion may be rare.

Larotrectinib was evaluated across 15 tumor types in 159 patients with TRK fusion cancer, and 121 (79%, 95% CI 72–85) of 153 patients had an objective response [16]. Of the 153 patients, 24 (16%) had a complete response, 97 (63%) had a partial response, 19 (12%) had stable disease, and nine (6%) had progressive disease [16]. Furthermore, Waguespack et al. reported the results of pooled data from three phase I/II larotrectinib clinical trials [10]. This study included 29 patients with TRK-fusion-positive thyroid cancer who were treated with larotrectinib. The histology was PTC in 20 (69%) patients, FTC in two (7%), and ATC in seven (24%), and the objective response rate was 71% among 28 evaluable patients [10]. The clinical responses were as follows: 2 (7%) had a complete response, 18 (64%) had a partial response, 4 (14%) had stable disease, and 3 (11%) had progressive disease [10]. The median response was 1.87 months [10]. Treatment-related adverse events (AEs) were reported in 26 patients (90%), the most common being myalgia, fatigue, dizziness, and elevated liver transaminase levels. Most treatment-related AEs were grade 1 or 2 [10]. In our case, the time to response was approximately one month. Furthermore, grade 1 myalgia, which occurred two weeks after the initiation of larotrectinib, was the only adverse effect. Therefore, larotrectinib treatment was effective and treatment-related AEs were tolerable.

To our knowledge, only three case reports of larotrectinib treatment for thyroid cancer have been published. Pitoia reported the case of a PTC patient with *ETV6-NTRK3* gene fusion who had previously been treated with sorafenib and lenvatinib [17]. Surprisingly, the patient achieved a durable (sustained for 11 months) complete response after 2 months of larotrectinib treatment and a complete intracranial response in metastatic brain lesions after 7 months of larotrectinib treatment [17]. Saliba et al. reported a case of secretory carcinoma in a patient with *ETV6–NTRK3* fusion [18]. Larotrectinib was administered for lung and neck metastases, and a durable response lasting 18 months was achieved [18]. Bargas et al. reported a case of PTC with *SQSTM1–NTRK* gene fusion [19]. Although the patient had hemoptysis and respiratory distress syndrome with marked dyspnea, all respiratory and thoracic symptoms disappeared two weeks after the initiation of larotrectinib [19]. In addition, the first CT evaluation after 2.5 months confirmed a partial response [19]. Despite our patient's history of mTKI treatment, larotrectinib was effective for distant metastases, similar to two previous case reports of patients with PTC. Therefore, larotrectinib is considered an effective treatment option even for patients with a history of mTKI treatment.

Distant metastases from differentiated thyroid carcinoma (DTC) mainly involve the lungs and bones, and metastases to other sites, such as the liver, are extremely rare [6]. Yoon et al. investigated 38,772 DTC patients and revealed that liver metastasis was observed in only five patients [6]. Although treatment outcomes have not been fully investigated among thyroid cancer patients with liver metastasis, TKIs may be beneficial [20, 21]. In our case, liver metastases were reduced by larotrectinib, which is a good treatment option for patients with TC and liver metastases.

Although the companion diagnostic test for larotrectinib is the FoundationOne CDx (Foundation Medicine Inc., Cambridge, MA, USA), the OncoGuide™ NCC Oncopanel System was performed in our case because the remaining liver biopsy specimens were small. The OncoGuide™ NCC Oncopanel System can generally be performed using fewer specimens than the FoundationOne CDx. In general, it is recommended that a specimen obtained within 3 years be used for genetic testing [22]. In this case, we performed effective genetic testing using fresh specimens.

In our case, the Tg levels decreased immediately after the initiation of larotrectinib. However, the patient was positive for TgAb, which might have affected the Tg levels. In general, an increase or reappearance of TgAb during follow-up is highly suggestive of recurrence or persistence [23]. In contrast, TgAb levels were reported to be correlated with the clinical response to lenvatinib [24]. The mechanism by which the TgAb level was positive after larotrectinib treatment is unknown. However, the TgAb level gradually decreased and liver metastases continued to shrink. Further studies are needed to investigate whether this response is specific to larotrectinib.

## Conclusion

In our case, larotrectinib demonstrated effective antitumor activity against liver metastases of PTC, a relatively rare site of distant metastasis. Furthermore, the efficacy of larotrectinib was maintained, even though the patient had a history of mTKI treatment and a relatively infrequent fusion gene, *SQSTM1–NTRK1*.

## Data Availability

The datasets used and/or analyzed during the current study are available from the corresponding author upon reasonable request.

## Abbreviations

AE: Adverse event
ALT: Alanine aminotransferase
AST: Aspartate aminotransferase
ATC: Anaplastic thyroid carcinoma
BUN: Blood urea nitrogen
CGM: Cancer genomic medicine
CGP: Comprehensive genomic profiling
Cre: Creatinine
CT: Computed tomography
DTC: Differentiated thyroid carcinoma
FTC: Follicular thyroid carcinoma
F-T4: Free thyroxine
F-T3: Free triiodothyronine
HCC: Hurthle cell carcinoma
MTC: Medullary thyroid carcinoma
NTRK: Neurotrophic-tropomyosin receptor kinase
PTC: Papillary thyroid carcinoma
PDTC: Poorly differentiated thyroid carcinoma
RAI: Radioactive iodine
Tg: Thyroglobulin
TgAb: Thyroglobulin antibody
TKI: Tyrosine kinase inhibitor
TSH: Thyroid-stimulating hormone
